# Monitoring Long-Term Waste Volume Changes in Landfills in Developing Countries Using ASTER Time-Series Digital Surface Model Data

**DOI:** 10.3390/s25103173

**Published:** 2025-05-17

**Authors:** Miyuki Muto, Hideyuki Tonooka

**Affiliations:** Graduate School of Science and Engineering, Ibaraki University, Hitachi 3168511, Japan

**Keywords:** remote sensing, ASTER, open dump, landfill, waste, digital elevation model, digital surface model, volume, monitoring, time-series analysis

## Abstract

Monitoring the amount of waste in open landfill sites in developing countries is important from the perspective of building a sustainable society and protecting the environment. Some landfill sites provide information on the amount of waste in reports and news articles; however, in many cases, the survey methods, timing, and accuracy are uncertain, and there are many sites for which this information is not available. In this context, monitoring the amount of waste using satellite data is extremely useful from the perspective of uniformity, objectivity, low cost, safety, wide coverage area, and simultaneity. In this study, we developed a method for calculating the relative volume of waste at 15 landfill sites in six developing countries using time-series digital surface model (DSM) data from the satellite optical sensor, the Advanced Spaceborne Thermal Emission and Reflection Radiometer (ASTER), which has accumulated more than 20 years of observational data. Unnecessary variations between images were reduced by bias correction based on a reference area around the site. In addition, by utilizing various reported values, we introduced a method for converting relative volume to absolute volume and converting volume to weight, enabling a direct comparison with reported values. We also evaluated our method compared with the existing method for calculating changes in waste volume based on TanDEM-X DEM Change Map (DCM) products. The findings of this study demonstrated the efficacy of the employed method in capturing changes, such as increases and stagnation, in the amount of waste deposited. The method was found to be relatively consistent with reported values and those obtained using the DCM, though a decrease in accuracy was observed due to the depositional environment and the absence of data. The results of this study are expected to be used in the future for technology that combines an optical sensor and synthetic aperture radar (SAR) to monitor the amount of waste.

## 1. Introduction

The increase in waste produced by human activities has a negative impact on society and the environment. According to the World Bank’s International Finance Cooperation (IFC), global waste generation has reached more than 2 billion tons per year and may increase by 70% by 2050 [1]. Proper waste management is essential for achieving a sustainable society and is related to many of the Sustainable Development Goals (SDGs). However, developing countries in particular face many problems in the operation of waste collection and accumulation facilities, and in many cases, inappropriate disposal methods such as open burning, landfilling, and dumping are employed, which reportedly account for approximately 69.7% of the global waste volume [1]. For example, India, with 17.8% of the world’s population, is predicted to become the world’s fourth largest waste generator by 2025 [2]. In India, about 50% of the collected waste is sent to landfills. India’s largest cities, Mumbai and Delhi, do not have sanitary landfills and rely on open dumping of municipal waste [3]. The World Bank reports that landfilling is one of the most common methods of municipal solid waste disposal worldwide [1]. Delhi has three landfills that became operational between 1984 and 1994, of which the Bhalswa and Ghazipur landfills exceeded their waste disposal capacity in 2002 and the Okhla landfill in 2010, but they have remained in use since then [4,5,6,7]. Thus, some landfills continue to be used even after exceeding their original capacity for 10 or 20 years. Improper landfill management through such open dumping has caused pollution problems in the surrounding environment (air, water, and soil) due to the leaching of hazardous chemicals. For example, landfill gasses (LFGs) are generated during the microbial biodegradation of organic matter contained in landfill waste or when compounds in the waste volatilize [8,9]. These gasses consist mainly of carbon dioxide, produced in an aerobic environment with high oxygen content, and methane, produced in an anaerobic environment with low oxygen content [4], and are factors in the generation of greenhouse gasses. These gasses also include sulfides, aromatics, and other gasses with unpleasant odors, which have been reported to pose a health hazard to nearby residents [10,11]. In addition, when rainwater percolates into the waste, leachate containing pollutants such as dissolved organic matter (DOM), inorganic salts, ammonia, heavy metals, and xenobiotic organic compounds (XOCs) is formed, contaminating groundwater [12] and adversely affecting the soil environment [13]. Therefore, the accurate monitoring of waste volumes in open dumps is an important step toward the proper management of landfills and the protection of the environment.

Methods for observing the quantity of various stockpiles, such as waste and gravel fills in landfills, can be divided into two categories: field survey methods and remote sensing (RS) methods. The former methods include invasive techniques such as electrical and electromagnetic surveys and borehole surveys [14,15,16,17,18] and non-invasive 3D measurements [19,20] using real-time kinematics (RTK), electronic total stations, and global navigation satellite system (GNSS) receivers. In particular, invasive methods have the advantage of delineating the boundaries between natural landforms and sediments and providing detailed and highly accurate data. However, in addition to the cost of preparing specialized equipment, these field survey methods require measurement at multiple points, which is time-consuming and costly in terms of time and manpower, especially for complexly shaped surfaces or large areas. Furthermore, there are health risks associated with working in a hazardous environment such as the risk of collapse, etc. [21,22]. Contrarily, the latter methods include 3D measurements using unmanned aerial vehicles (UAVs) and ground-based LiDAR [21,23,24,25,26,27,28,29,30,31,32,33,34], aerial photography [35], satellite synthetic aperture radar (SAR) images [36,37,38,39], satellite optical images [40], etc. Although these RS methods are often less accurate than field surveys, except for 3D measurements using UAVs and ground-based LiDAR, they are superior in terms of cost and risk and are therefore suitable for repetitive landfill monitoring applications. Indeed, in methods using RS, UAV photogrammetry has proven effective in a variety of environments, including landfills, because it provides accurate volumetric measurements [29]. In a review article on the use of UAV technology in waste disposal site (WDS) management [27], it was reported that 48% (33 publications) of the studies published between 2010 and 2020 addressed the spatial characteristics of WDSs (including waste mass parameters such as height, slope angle, surface area, and disposal volume). However, the use of satellites is superior to that of UAVs for the simultaneous, long-term monitoring of large areas and numerous landfills.

Regarding the survey of wastes using RS, including ocean dumping, there were reported cases of the use of aircrafts and satellites in the 1970s [41,42], and these techniques are now used not only to monitor waste but also in the entire life cycle of landfills, including landfill area selection and environmental assessments [37]. Studies have also been conducted to estimate the volume of waste at a given point in time or to estimate changes in waste volume within a certain time period for a particular landfill [21,23,24,25,26,35,36]. In these studies, satellite optical imagery has the advantage of being highly interpretable, although the availability of observation is weather dependent. Many studies on landfills using such imagery have focused on the extent of surface area of landfills and the detection of waste materials [37]. However, there are few studies on the thickness and volume of sediments in landfills, such as the case of Sasaki et al. (2006), who obtained original stream elevations from shadows in a 1988 SPOT ortho image to determine the amount of illegally dumped waste in the form of filling mountain streams [40]. Contrarily, satellite SAR has a significant advantage, as it can be used for constant monitoring regardless of weather conditions; moreover, interferometric SAR is effective, as it can determine the degree of landfill variability. For example, Karathanassi et al. (2012) created 13 ENVISAT ASAR pairs from a dataset of 11 images of a landfill in Athens, Greece, taken between 2003 and 2004, and applied repeat-path SAR interferometry to generate digital elevation models (DEMs) of the landfill at two different times using the ENVISAT ASAR method to analyze changes in waste volume based on the deformations between them [36]. While this showed that changes in waste volume and areas of active waste disposal activity could be effectively displayed through elevation profile lines, problems with estimation accuracy were noted due to the orientation of the landfill area and weather conditions. Du et al. (2021) used Sentinel-1A, Radarsat-2, ALOS-2, and TerraSAR-X/TanDEM-X images obtained from 2013 to 2020 to analyze the relationship between landfill (Xingfeng Landfill (XFL)) subsidence and landfill thickness [38]. Thus, the use of satellite SAR is effective in analyzing landfills, but it should be noted that it is not as good as optical sensors in terms of interpretability, temporal correlation removal and phase unwrapping in InSAR are difficult in areas that change sequentially like landfills [43], and that there are problems unique to SAR such as shadowing.

As demonstrated above, satellite RS has been shown to be a beneficial technology for the monitoring of waste. Nevertheless, there is a paucity of studies that have estimated changes in waste volume at multiple landfill sites around the world using satellite images, with the exception of the work of Louw et al. (2025) [44]. They used the Tandem-X DEM Change Map (DCM), a DEM dataset, to determine the change in waste volume between two time points between 2018 and 2021 for up to 1177 days (3.2 years) for landfills in 100 countries worldwide. However, the time difference between the DCM maps at the two time points must be sufficient to estimate the change in landfill volume, and if this condition is not met, it is difficult to estimate the change in landfill volume. In fact, 24% of the total sites did not have sufficient observational data, and for more than 30% of the sites, the authors reported that the observation period between the two time points was less than 50 days. Another issue is that the evaluation of the time period is limited to only two time points.

Against this background, this study develops a method for analyzing the amount of waste in landfill sites over time using digital surface model (DSM) images from the satellite optical sensor ASTER (Advanced Spaceborne Thermal Emission and Reflection Radiometer), which has an archive of over 20 years and has stereo observation capabilities, and applies it to 15 landfill sites in six developing countries. There are several studies that have analyzed topographical deformation caused by sedimentation, etc., by obtaining the difference in DSM between different points in time through 3D measurement using optical sensors mounted on satellites and drones [43,45,46,47,48], but to the best of our knowledge, there are no studies that have analyzed the long-term changes in the amount of waste at multiple waste landfill sites. The number of images that can be acquired is limited depending on the climatic conditions where the site is located, which is a disadvantage, but it is expected that it will be possible to evaluate multiple landfill sites using a common method and to evaluate the time variation in waste volume over a longer period and at a higher frequency than with the method using the DCM at two points in time presented by Louw et al. (2025) [44]. 

## 2. Materials and Methods

### 2.1. Study Sites

**Figure 1:**
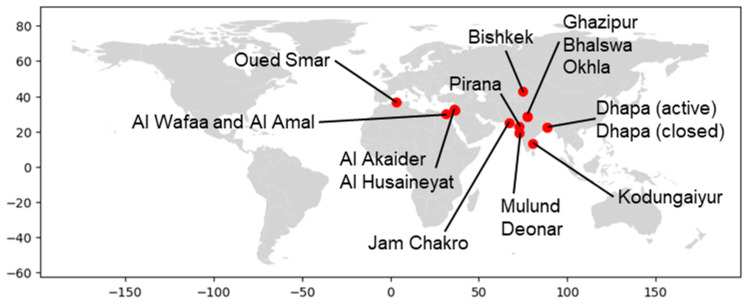
The locations of the 15 open dump sites selected as study sites.

**Figure 2:**
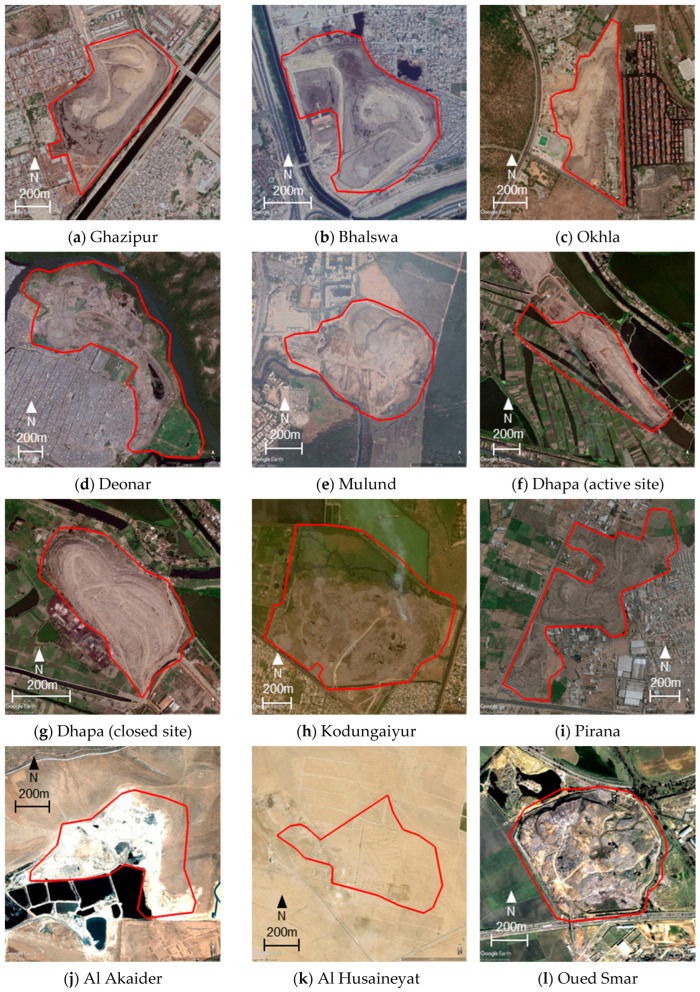

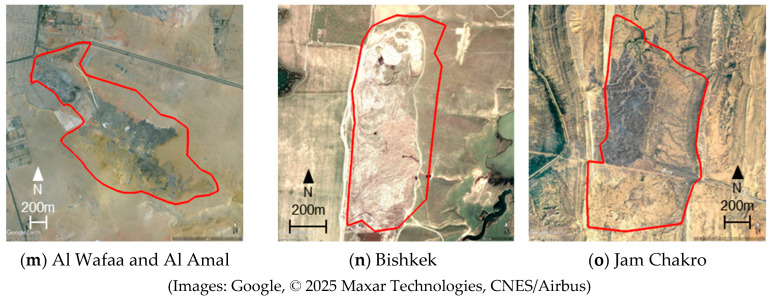
The outer edges of each site shown on Google Earth Images for 15 sites. The geolocations of each image are listed in Table 1. Image acquisition dates are as follows: (**a**) 10 June 2010, (**b**) 13 June 2010, (**c**) 10 June 2010, (**d**) 3 November 2009, (**e**) 28 January 2011, (**f**) 1 March 2011, (**g**) 1 March 2011, (**h**) 5 April 2011, (**i**) 27 April 2012, (**j**) 8 June 2004, (**k**) 1 January 2004, (**l**) 16 February 2000, (**m**) 11 June 2003, (**n**) 2 May 2004, and (**o**) 30 January 2004.

In this study, a total of 15 open dump sites in six developing countries were selected as study sites. Figure 1 shows the locations of the 15 sites. Table 1 shows the names, countries, geographical coordinates, estimated areas, start-up years, and operational status of each site. All of these sites are landfill sites located in the suburbs of cities in developing countries, and information on the amount of waste was available on the Internet in the form of news articles and reports. Of the 15 sites, 9 were selected from India. This is because India has the world’s largest population, there are many large open landfill sites in the suburbs of cities, and English-language literature is easy to obtain. The other six sites were selected as follows: two sites from Jordan and one site each from Algeria, Egypt, Kyrgyzstan, and Pakistan. In the site selection process, we avoided sites that involved the reclamation of water bodies, and we also needed the site to have at least three available clear ASTER images. Figure 2 shows the Google Earth Pro images for each site, with the red lines indicating the boundaries of each site. When defining these boundaries, we used Google Earth images that were taken after the start of landfill operations and were clear and as old as possible as base images, and we also used literature and new images related to each landfill as reference information. When defining boundaries, areas where buildings or other structures were confirmed to exist were excluded from the analysis area as much as possible, while areas that were originally flat but later expanded as landfill sites were included in the analysis area as much as possible. Therefore, some sites in Figure 2 include areas where there was no waste at the time the base image was acquired, as well as areas that were excluded due to the presence of buildings, etc. The areas intentionally included or excluded in this manner are small parts of each site, and since they are located near the boundaries away from the central parts of the sites where waste normally accumulates in large quantities, their impact on the analysis is not expected to be significant. The areas in Table 1 are estimated values based on the respective outer boundaries.

Of the nine sites in India, Ghazipur, Bhalswa, and Okhla are the main landfill sites in the capital city of Delhi [49]. Deonar and Mulund are located in the suburbs of Mumbai, with Deonar being the largest landfill site in the city and Mulund the second largest [50]. Dhapa is a landfill site in Kolkata. The closed and operational areas are adjacent to each other, and in this study, they are treated separately as a closed site and active site, respectively. Deonar is a landfill located in the eastern suburbs of Mumbai. Kodungaiyur is located in the northern part of the Chennai metropolitan area. Pirana is located in the south of Ahmedabad.

Of the six sites outside India, Al Akaider and Al Husaineyat are landfill sites in Jordan. The former is located in the southeast of Al-Ramtha and is the second-largest landfill site in Jordan. The latter is located 18 km southeast of the city of Mahraq. Oued Smar is a landfill located in the southeastern suburbs of Algiers, the capital of Algeria. Al Wafaa and Al Amal are a single site located between Cairo and New Cairo in Egypt. Bishkek (Bishkek Solid Waste Landfill (BSWL)) is located in Leninskoe, which is adjacent to the north of Bishkek, the capital of Kyrgyzstan. Jam Chakro is a landfill located about 20 km north of the center of Karachi, Pakistan.

**Table 1:** Location, area, operation status, and other information for 15 study sites.

Site Name	Country	Location (Lat., Long.)	Estimated Area (km^2^)	Start Year of Operation	Operation Status	References
Ghazipur	India	28.624, 77.327	0.31	1984	–	[51,52,53]
Bhalswa	India	28.740, 77.156	0.27	1992–1994	–	[53,54]
Okhla	India	28.512, 77.284	0.23	1994–1996	Biomining started in July 2019	[53,55,56,57,58]
Deonar	India	19.072, 72.928	1.31	1927	–	[59,60,61]
Mulund	India	19.170, 72.973	0.33	1967–1968	Closed in October 2018	[62,63,64]
Dhapa (active site)	India	22.536, 88.425	0.53	1987	–	[65]
Dhapa (closed site)	India	22.544, 88.419	0.16	1987	Closed in 2009	[65,66]
Kodungaiyur	India	13.136, 80.268	1.22	1987	–	[67,68]
Pirana	India	22.980, 72.567	0.42	1980	–	[69,70]
Al Akaider (Al-Ekaider)	Jordan	32.514, 36.111	0.42	1980–1981	–	[71,72,73]
Al Husaineyat (Mafraq FDS)	Jordan	32.255, 36.349	0.35	1986	–	[71]
Oued Smar	Algeria	36.698, 3.155	0.46	1978	Closed in 2011	[74,75]
Al Wafaa and Al Amal (El-wafaa and El-amal)	Egypt	30.017, 31.362	2.66	Late 1970s	Closed in 2018 or 2019	[15,76]
Bishkek (BSWL)	Kyrgyzstan	42.968, 74.590	0.49	1974–1976	–	[77,78]
Jam Chakro	Pakistan	25.030, 67.032	1.22	1996	–	[73]

### 2.2. Data Used

This study used the ASTER DSM images from the ASTER Value-Added (VA) product [79], which are freely available from the METI AIST Data Archive System (MADAS), operated by the Ministry of Economy, Trade and Industry (METI) and the National Institute of Advanced Industrial Science and Technology (AIST), Japan. ASTER is a medium-resolution multispectral sensor onboard the NASA’s Terra satellite and consists of three subsystems divided with respect to the wavelength range: visible and near-infrared (VNIR), short-wave Infrared (SWIR), and thermal infrared (TIR) subsystems. Each subsystem has 3, 6, and 5 spectral bands, respectively, and spatial resolutions of 15, 30, and 90 m, respectively. Band 3 in the near-infrared region of the VNIR radiometer can observe two directions, nadir and backward, and the stereo imaging method combining these data can generate a DEM, where this DEM product is strictly a DSM and retains the height of its upper edge for buildings and vegetation. In this study, we refer to the product name as DEM, but to avoid confusion, we use the term DSM in other places. The DEM product provided through MADAS has a resolution of 30 m and is geoid corrected to zero at sea level using the Earth Gravitational Model 1996 (EGM96). The accuracy of the DEM product is reported to have a maximum surface pixel position error of 50 m and a maximum elevation error of less than 15 m [80].

First, for each study site, ASTER DSM images that were observed between 2000 and 2024 and had a cloud cover of 10% or less were obtained. Then, from this dataset, only the images that had no clouds at or near the site by visual interpretation were selected. The number of selected images was highly dependent on the clear sky ratio of each site, ranging from 6 images (Deonar, India) to 37 images (Al Wafaa and Al Amal, Egypt).

**Figure 3:**
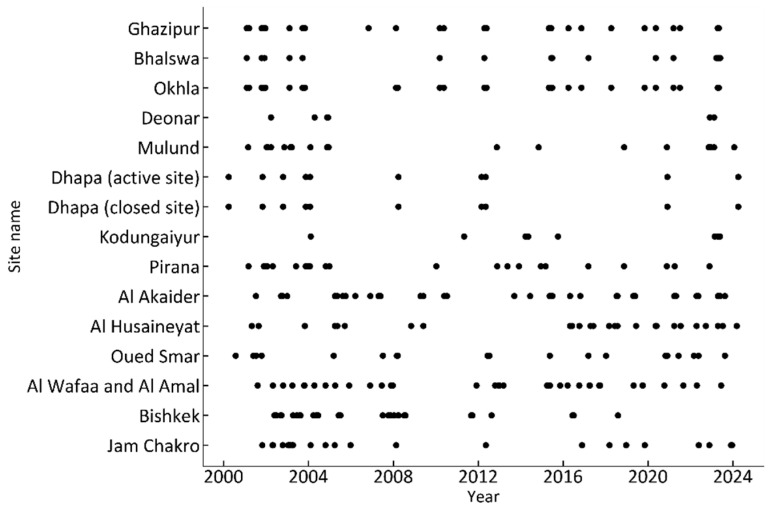
Distribution of observation dates for ASTER DSM images of each site.

**Figure 4:**
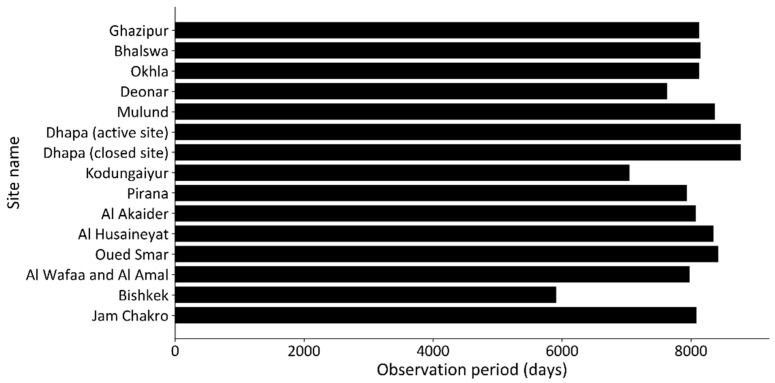
Observation period for each site (number of days from observation date of oldest image to observation date of newest image).

Figure 3 shows the distribution of the observation dates for each image at each site, with the vertical axis representing each site and the horizontal axis representing the observation date (year). As shown in this figure, the observation interval for images at each site is not constant, as it is affected by the observation scheduling of ASTER as well as the rate of clear days at the site. The longest period without images during the observation period is 18 years (6571 days) at the Deonar site. Figure 4 shows the observation period (the number of days from the observation date of the oldest image to the observation date of the newest image) for each site, with the vertical axis representing each site and the horizontal axis representing the observation period at each site. The observation period differs depending on the site, ranging from 16 to 24 years. Table 2 shows the observation date and time of the oldest image and the newest image for each site.

### 2.3. Method

#### 2.3.1. Estimating Amount of Waste Using ASTER DSM Images

**Figure 5:**
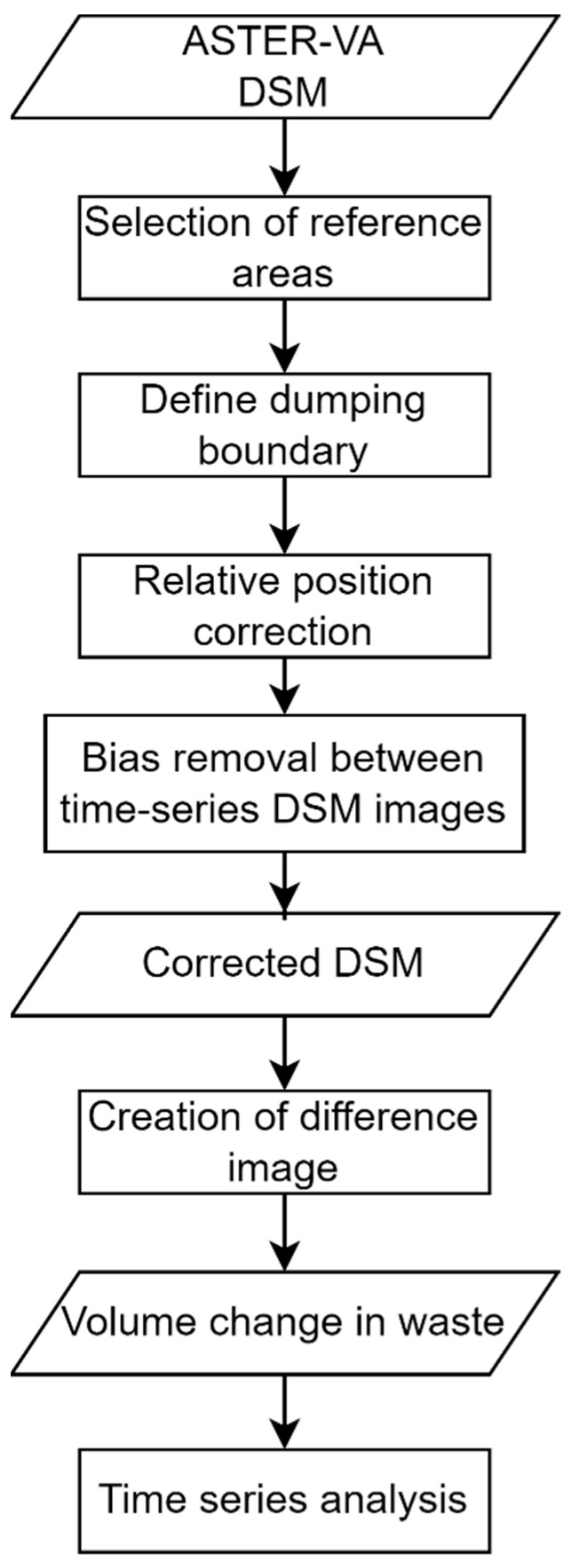
Flow of estimating the relative volume of waste using ASTER DSM images.

The flow for estimating the relative volume of waste using the ASTER DSM images is shown in Figure 5. This process is broadly divided into three steps: (1) position correction, (2) height correction, and (3) estimation of the relative volume of waste. The details of each step are described below.

(1) Position correction

It has been reported that the maximum ground pixel position error in the ASTER DSM images is 50 m [80]. Such position errors are a major source of error in waste volume assessments based on the time-series analysis. Therefore, we corrected the positional errors so that those between the ASTER DSM images used at each site were within 1 pixel. Since it is difficult to determine the ground control points from the DSM images, we evaluated the positional errors at the ground control points using the ASTER Band 3N images used to generate the DSM images and corrected the positional errors by a parallel shift if they were 2 pixels or more.

(2) Height correction

As a result of preliminary research on time-series DSM images at several sites, we found cases where a difference of up to 30 m was seen in the DSM values between time-series images, even in areas where there appeared to be no change in land use in the vicinity of the site. Such differences are thought to be due to factors such as ASTER line-of-sight vector errors and stereo matching errors, and they are a significant source of error in the analysis of the volume of waste in this study. Therefore, height correction was carried out for each DSM image using the following procedure.

First, at each site, we selected at least five reference areas within a few kilometers of the landfill site that had not changed in land use during the time period of the time-series images using Google Earth Pro images and ASTER simulated true-color images. These reference areas were 3 × 3 pixels or larger. Next, the oldest image in the time-series image set was designated as the reference image A, and the image to be height-corrected was designated as the target image B. For each reference area at each site, the difference between the area-averaged DSM value of the target image B and the area-averaged DSM value of the reference image A was calculated. Thus, if the area-averaged DSM values of images A and B in the reference area *r* are $\bar{Z_{A,r}}$ and $\bar{Z_{B,r}}$, respectively, the difference in DSM values in the reference area *r*, $\Delta Z_{r}$, between images A and B is expressed by the following formula:

(1) $$\Delta Z_{r}=\bar{Z_{B,r}}-\bar{Z_{A,r}}$$

Next, the median of $\Delta Z_{r}$ was calculated between the reference areas, and this was taken as the bias value of the target image B with respect to the reference image A.

(2) $$bias=\mathrm{median}\left(\Delta Z_{r}\right),\;\;r=1,\;2,\;\cdots ,\;N$$

where *N* is the number of reference areas. Then, the height of image B was corrected by subtracting this bias value from the total DSM value of image B.

**Figure 6:**
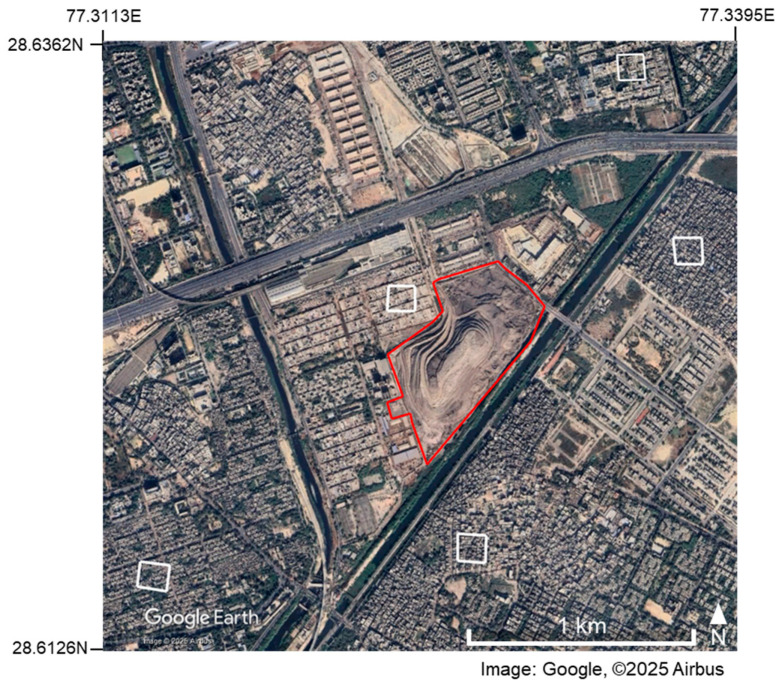
Five reference areas selected at the Ghazipur site, as shown on a Google Earth image acquired on 4 December 2024.

**Figure 7:**
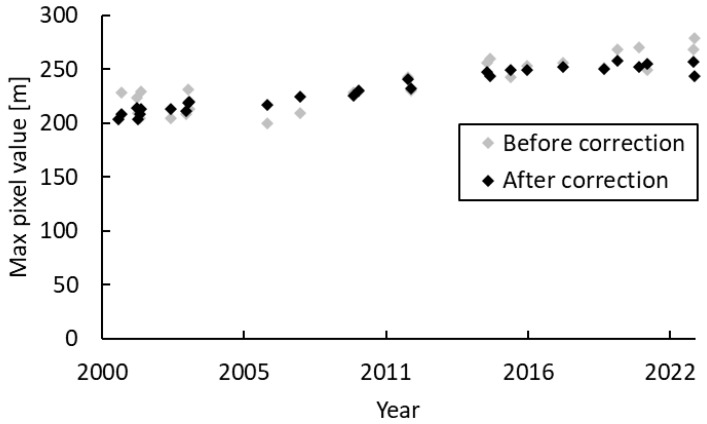
Time-series changes in maximum DSM values before and after height correction at the Ghazipur site.

Figure 6 shows the five reference areas selected for the Ghazipur site (the area surrounded by a red line) with white frames. Figure 7 compares the time-series changes in the maximum DSM values at the same site before and after height correction. The vertical axis shows the maximum DSM value (m) in the site area in the images taken at each time, and the horizontal axis shows the observation date (gray diamond: before correction; black diamond: after correction). The site shows an increasing trend in the time series after height correction during this period.

(3) Estimation of the relative volume of waste

In order to calculate the absolute volume of waste material from each DSM image at each site, topographical data for when there was no waste material are required. However, as shown in Table 1, all of the sites have been in operation since before the start of ASTER observations; thus, such initial topographical data cannot be obtained from ASTER products. Therefore, in this study, the oldest image at each site was used as the reference image A, and the relative volume of waste was calculated from the difference in DSM values between the target image B and reference image A.

First, for pixel *k* in site S, the DSM value of target image B was set as *Z_B_*_,*k*_, and the DSM value of reference image A was set as *Z_A_*_,*k*_ (unit: m). The difference between these values was then calculated for all pixels in site S and then multiplied by the area of a single pixel (900 m^2^ (=30 m × 30 m)) to obtain time-series data for the relative volume of waste *V_rel_*, with the reference image A serving as the standard.

(3) $$V_{rel}=\underset{k\in S}{\sum }\left(Z_{B,k}-Z_{A,k}\right)\times 900$$

Subsequently, a regression analysis was conducted, with the number of days that had elapsed since the observation date of the reference image A serving as the explanatory variable, resulting in a quadratic polynomial regression curve.

(4) $$V_{rel}\left(D\right)=a\times D^{2}+b\times D+c$$

In this equation, *a*, *b*, and *c* represent the regression coefficients. It is noted that linear regression (*a* = 0) was employed solely for these two sites due to the unavailability of enough images for Deonar and the substantial variation observed for Jam Chakro. Subsequently, to account for the inaccuracies inherent to the DSM values of the reference image A, the offset *c* of the obtained regression equation was subtracted from *V_rel_* values, and the regression estimate of the relative volume of waste after offset correction at elapsed day *D*, *V*′*_rel_*(*D*) (unit: m^3^), was obtained.

(5) $${V^{\prime }}_{rel}\left(D\right)=a\times D^{2}+b\times D$$

In this equation, *a* = 0 for Deonar and Jam Chakro. Additionally, the regression estimate for the reference image A (*D* = 0) is 0 at all sites.

(4) Calculation of the absolute volume of waste

If we assume the initial topography before the waste was brought to each site, we can calculate the absolute volume of the waste. Let *Z*_0,*k*_ be the initial elevation at pixel *k* at site S. In this case, the absolute volume of the waste in the reference image A, *V_abs,A_*, can be calculated using the following formula.

(6) $$V_{abs,A}=\underset{k\in S}{\sum }\left(Z_{A,k}-Z_{0,k}\right)\times 900$$

Then, by adding this value to *V*′*_rel_*(*D*), the absolute volume of waste *V_abs_*(*D*) at elapsed day *D* can be calculated.

(7) $$V_{abs}\left(D\right)={V^{\prime }}_{rel}\left(D\right)+V_{abs,A}$$

The absolute volume of waste obtained in this way is less reliable than the relative volume of waste because it includes assumptions about the initial topography.

For the initial elevation *Z*_0,*k*_ for each site, this study used a simple method that assumed the flatness of each site, extracted pixels that were two pixels outside the area boundary pixels of each site, and used the average of the DSM values of these pixels as the initial elevation value for all pixels within the site. A similar approach is described by Jiang et al. (2022) as a method for determining the base height when estimating the amount of debris using a drone [48].

#### 2.3.2. Evaluation of ASTER-Based Values Using Reported Values

Information on the amount of waste at the 15 sites selected for this study is publicly available in the form of papers, reports, news articles, etc. We used this information to evaluate the effectiveness of waste monitoring using the ASTER DSM images. This evaluation was conducted using the following three methods, taking into account the fact that the type and reliability of the publicly available information varies.

(1) Evaluation based on total amount using annual waste amount information

At some sites, the total weight (in tons) of waste brought in each year is reported for the entire period of operation or for part of the period. At these sites, the absolute volume of waste at the ASTER reference date (the observation date of reference image A) was estimated by finding the ratio of the total weight between the period from the start of operation to the ASTER reference date and the period from the ASTER reference date to a certain point in time, assuming that the total weight ratio and total volume ratio are equal under the assumption that the waste density does not change during the period of operation. This was then added to the ASTER time-series relative volume data to convert it into time-series absolute volume data. In addition, because the density of the waste was reported for some sites, the time-series absolute volume data was multiplied by the density to obtain time-series weight data, and an evaluation was carried out based on weight. In this way, the total amount of waste reported at a certain point in time was directly compared with the total amount of waste based on the ASTER data for each site.

(2) Evaluation based on total amount, assuming initial topography of the site

The ASTER-based time-series absolute volume data can be obtained under the assumption of the initial topography of the site using the procedure in Section 2.3.1 (4). Multiplying these by the density yields the time-series absolute weight data. As the initial topography is assumed, the reliability of these data is low, but this method allows for the direct comparison of the total amount of waste at a certain point in time, described in many reports, with the total amount of waste based on ASTER.

(3) Evaluation based on the amount of change over a certain time period

Some reports indicate the amount of waste brought in per day or per year. These values can also be calculated from the regression equation for the time-series relative volume data based on ASTER obtained in the procedure in Section 2.3.1 (3). Therefore, we directly compared the reported values and the ASTER-based values based on the amount of change over a certain time period.

#### 2.3.3. Evaluation Based on Volume Change Between Two Time Points Using DCM Products

As in the study by Louw et al. [44], the change in the volume of waste between two times at each site was estimated from the Tandem-X DEM Change Map [81] provided by the German Aerospace Center and compared with the same change obtained from the ASTER time-series relative volume data.

The resolution of the DCM dataset in the latitude direction is 1 s (approximately 31 m), and the resolution in the longitudinal direction depends on the latitude but is 30.92 to 19.88 m for the sites in this study, all of which are below 50 degrees latitude. For latitudes between 0 and 60 degrees, a range of 1 degree latitude by 1 degree longitude is defined as one tile, and two change maps (DCM_1_ and DCM_2_) are provided for each tile. These change maps all represent changes between the TanDEM-X 30 m Edited DEM collected between 2016 and 2022 and the reference DEM, and in areas where multiple observations were made during the period, DCM_1_ and DCM_2_ differ, but in areas where only one observation was made, they are the same. In other words, in order to calculate the volume change between two points in time, it is necessary for TanDEM-X to have made multiple observations of that site during the period. After investigating the 15 sites in this study, it was found that only 6 sites (Bhalswa, Okhla, Deonar, Mulund, Kodungaiyur, and Bishkek) had multiple TanDEM-X observations between 2016 and 2022, and two different change maps were provided; thus, the volume change in waste was calculated for these 6 sites using the DCM product according to the following procedure.

1. In the rectangular area, including the surrounding area of each site, the height change was calculated by subtracting the DCM_1_ value from the DCM_2_ value for each pixel.
2. For each pixel, if the absolute value of the height change obtained was the square root of the sum of the squares of the height accuracy indication (HAI) values or greater, included in the DCM product of DCM_1_ and DCM_2_, it was considered to be a valid pixel.
3. The height change value of each valid pixel was multiplied by the pixel area, divided by the number of days between DCM_1_ and DCM_2_, and the volume change per day for each pixel was calculated.
4. The volume change per day for waste at the site was calculated by taking the sum of the volume change per day of all valid pixels.

## 3. Results

### 3.1. Time-Series Plot of Relative Volume of Waste Obtained from ASTER DSM Data

**Figure 8:**
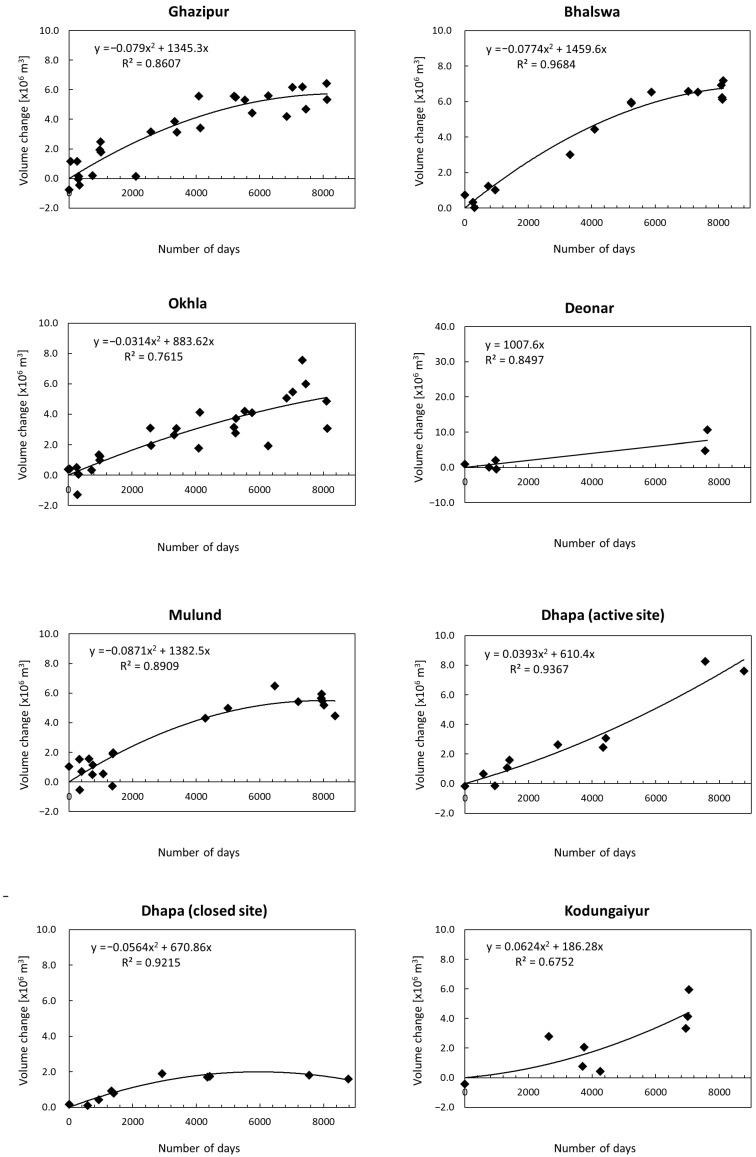

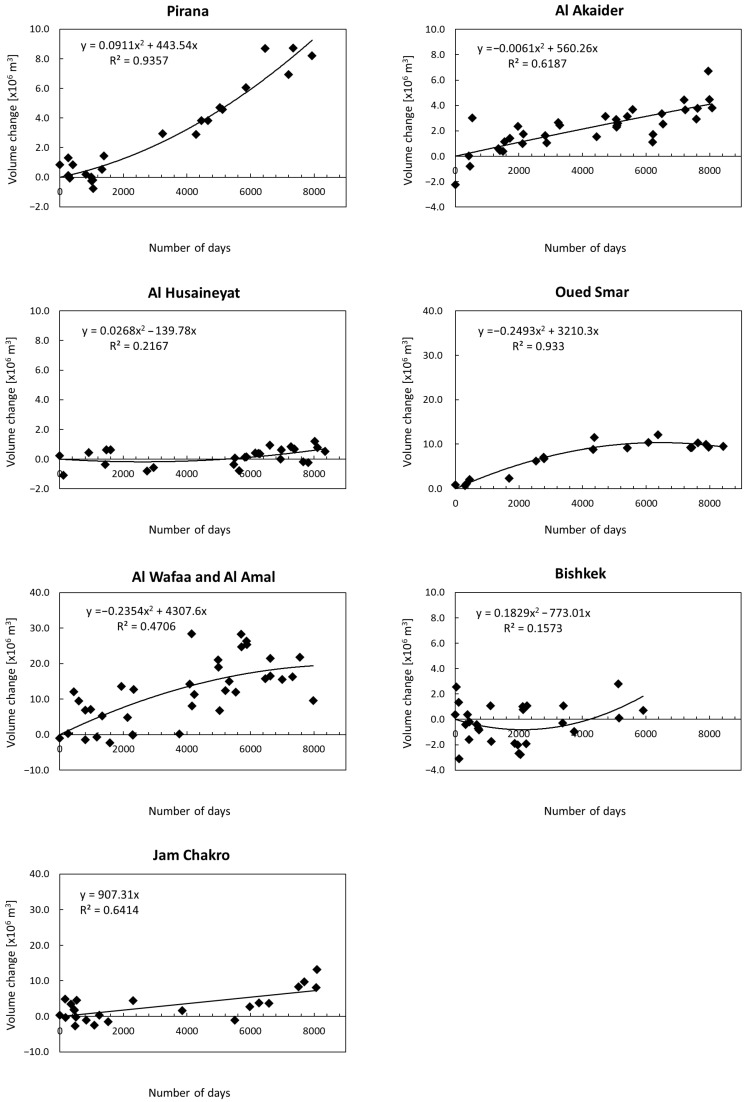
Time variation in the relative volume of waste obtained from ASTER time-series DSM data for each site.

Figure 8 shows the time-series changes in the relative volume of waste (volume change from the reference image date) obtained at each site. The horizontal axis shows the number of days that passed since the reference image date. The vertical axis shows the relative volume of waste, which is offset so that the regression curve passes through the origin. At all sites, the relative volume of waste is shown to increase over time. The coefficient of determination is somewhat low for the 3 sites of Al Husaineyat, Al Wafaa and Al Amal, and Bishkek, but the other 12 sites have a coefficient of determination of 0.5 or more. The results for each site in Figure 8 are described below.

The Ghazipur, Bhalswa, and Okhla sites, all of which are located in Delhi and are currently in operation, showed a trend of increasing relative volume throughout the observation period, but the rate of increase gradually slowed down. The Municipal Corporation of Delhi (MCD) began biomining using trommel machines at these sites in 2019 [49,82,83], and the timing of the gradual increase in speed (D = 6540 on 1 January 2019) is generally consistent. At Ghazipur, there is an image with a large residual from the regression curve at D = 2096; this image has a cloud cover of 1% in the metadata, but we have confirmed that there is a thin cloud covering the entire image, and this is thought to be the reason for the large residual.

At the Deonar site, only six images were used, and there was a 6571-day data gap in the middle of the observation period, with only two images taken after that. For this reason, the analysis results for this site are not very reliable, but they do show an increasing trend in relative volume.

At the Mulund site, the relative volume shows an increasing trend up to around D = 6000 but then begins to decrease. This is consistent with the fact that this site was closed in October 2018 (D = 6489 on 1 October 2018) [64].

The relative volume of the Dhapa (active) site has been increasing year by year. This change may be due to the increase in waste brought in following the closure of the adjacent Dhapa (closed) site in 2009 (D = 3200 on 1 January 2009) and the expansion work carried out at the site in 2010 (D = 3565 on 1 January 2010) [84]. Contrarily, the Dhapa (closed) site was closed in 2009 (D = 3200 on 1 January 2009), and news in 2017 stated that the mound at this site would be covered with sheets and turned into a garden for tourists by 2020 [85]. In fact, the plot shows that the relative volume has remained almost constant since around D = 3200.

The Kodungaiyur site was still in operation as of 2024 [86]. The plot also shows that the relative volume continues to increase.

The Pirana site was still in operation as of 2023 [87], but the plot shows that the increase in relative volume has stalled since 2019. This may be related to the fact that a trial project for biomining began in September 2020 (1 September 2020 was D = 7119) [88].

At the Al Akaider site, the relative volume shows an increasing trend. The coefficient of determination is 0.6187, but if we exclude two points (D = 537 and D = 7953), the coefficient of determination rises to 0.7303. The reason for the large residuals in these two images is that they are not clear.

The plot for the Al Husaineyat site shows a large amount of scatter, making it difficult to discern any trend in the change in relative volume. However, if we focus on the second half of the observation period, when there are more data points, we can see that the relative volume is increasing.

The Oued Smar site was closed in 2011 (D = 3810 on 1 January 2011) and is now an urban park [75]. The change in relative volume has also stagnated since that time.

The Al Wafaa and Al Amal site shows a large variation in values overall, but the increase in relative volume slows down around D = 5000–6000. This slowdown is consistent with the fact that operation of the site was suspended in 2019 (D = 6353 on 1 January 2019) [15].

The Bishkek and the Jam Chakro sites both show large fluctuations in the early stages of the observation period, but the former shows an increasing trend in the latter half of the observation period, and the latter shows an increasing trend throughout the observation period.

Table 3 shows the root mean square (RMS) of the regression residuals, the maximum relative change during the ASTER observation period, and the ratio of the former to the latter for each site. The RMS of residuals is below 1.5 (×10^6^ m^3^) at 11 sites, but 2 sites, the Dhapa (active) site and the Al Wafaa and Al Amal site, show large values of 3.890 and 6.509 (×10^6^ m^3^), respectively. The maximum relative change varies greatly between sites, with the minimum at the Al Husaineyat site (0.701 × 10^6^ m^3^) and the maximum at the Al Wafaa and Al Amal site (19.38 ×10^6^ m^3^), but 10 sites fall within the range of 4.1 to 9.3 (×10^6^ m^3^). The ratio is considered to indicate the reliability of ASTER’s relative change estimates, with lower values indicating higher reliability. All 10 sites have a ratio of 0.3 or less, indicating relatively high reliability, but the Al Husaineyat site and the Bishkek site exceed 0.7, indicating lower reliability.

### 3.2. Comparison of ASTER-Based Values and Reported Values Based on Total Waste Volume at Certain Points in Time

For Ghazipur, Bhalswa, Okhla, Deonar, Dhapa, and Pirana, which are located in India, we were able to obtain reported values for the amount of waste (volume or weight) per year, along with the ASTER reference date, so we used the method described in Section 2.3.2 (1) to calculate the absolute volume of total waste from the ASTER regression estimates at the date of the reported value, and these values are provided in the Section 2.3.2 (1) column of Table 4. The comparable reported values are also shown in Table 4. Here, for Dhapa, the reported value was the combined value for the active and closed sites, so both sites are shown together as “active + closed sites”. In addition, unlike for Ghazipur, Bhalswa, and Dhapa (active + closed sites), the total annual volume of waste was not reported for Okhla, Deonar, and Pirana, so the reported waste density was used to convert the weight to volume. For dates, if the date of the reported value was given in the report, that date was used; if only the year was given, the first day of that year was used; and if only the month and year were given, the first day of that month was used. If there was no mention of the date of acquisition of the reported value, the date of publication of the report was used.

As shown in the table, the difference between the ASTER-based values and the reported values is less than 30% for Ghazipur, Bhalswa, Okhla, and Dhapa, indicating a high level of consistency. The reported value for Pirana is shown as a range and is smaller than the ASTER value, but the upper limit is close to the ASTER value. On the contrary, the difference for Deonar is rather large, and the ASTER value is about half the reported value. As shown in Figure 8, Deonar only has six ASTER images, including the reference image, and the reliability of the regression equation is lower than for the other sites, so there is a possibility that the ASTER value is inaccurate.

The values in the Section 2.3.2 (2) column of Table 4 are the total amounts of waste based on ASTER, assuming the initial topography of each site using the method described in Section 2.3.2 (2). This method could be applied to sites such as Mulund and Kodungaiyur, which could not be analyzed using the method in Section 2.3.2 (1). However, the four sites of Al Akaider, Al Husaineyat, Oued Smar, and Jam Chakro were excluded from the evaluation because, although the reported values for weight were obtained, the reported values for volume and density were not obtained.

As shown in the table, the difference between the reported values (or parts of the reported values) and the ASTER values for eight sites (excluding Dhapa (active site), Al Wafaa and Al Amal, and Bishkek) is 30% or less. For Al Wafaa and Al Amal, there is a large variation in Figure 8, so it is highly likely that the ASTER values are inaccurate. The same is true for Bishkek. In contrast, the difference for Dhapa (active site) in 2014 was 93.5%, but in 2009, it was 25.9%, so there is a possibility that the problem lies with the reported values rather than the initial topography.

### 3.3. Comparison Between ASTER-Based Volume Change per Day and Reported Amount of Waste Disposed of per Day

Table 5 compares the ASTER-based daily volume change obtained using the method described in Section 2.3.2 (3) with the reported amount of waste disposed of per day. The ASTER-based values are the values on the ASTER reference date and the values on the most recent image observation date. The four sites of Al Akaider, Al Husaineyat, Oued Smar, and Jam Chakro were excluded from the evaluation because the reported values for the amount of waste disposed of over a certain period were not available. In addition, although we were able to obtain reported values for the amount of waste disposed of per day for the Al Wafaa and Al Amal site, we were unable to obtain reported values for density, so we excluded this site from the evaluation. For Bishkek, the reported value of 5.17 was used because it was reported as the volume of waste disposed of per day, but for the other sites, only the weight of waste disposed of was reported, so we used the reported values for density at each site to convert the weight to volume.

As shown in the table, the ASTER values for the two sites of Bhalswa and Mulund are within the range of the reported values, but for the other sites, with the exception of Bishkek, the ASTER values are slightly smaller than the reported values. Possible reasons for this include the effect of waste compression, the reduction in accumulated waste due to biomining and fires, etc. For Bishkek, there is a large discrepancy between the ASTER values and the reported values. As shown in Figure 8, the ASTER estimates for Bishkek have a large degree of variation, and the reliability of the regression equation is low, so it is thought that there is a problem with the ASTER-based values.

### 3.4. Comparison of DCM-Based and ASTER-Based Values for Volume Change Between Two Time Points

The processing described in Section 2.3.3 was carried out for the six sites (Bhalswa, Okhla, Deonar, Mulund, Kodungaiyur, and Bishkek), where two different DCMs were provided between 2016 and 2022. The results obtained are shown in Table 6.

As shown in Table 6, the shortest period between DCM_1_ and DCM_2_ is 473 days for Bishkek, and the longest is 957 days for Okhla, with an average of 794.8 days for the six sites. Furthermore, the minimum valid pixel ratio is 51.1% for Deonar, and the maximum is 94.5% for Okhla, with an average of 68.7% for the six sites. As such, it should be noted that the results of the DCM have a short analysis period of 1–3 years and that there are many interpolated values within the site.

The table displays the estimated values for volume change per day using the DCM, the estimated values obtained from the regression formula for ASTER for the same period as the DCM (the period from DCM_1_ to DCM_2_), and the difference between these. Since the DCM value was calculated using only valid pixels, it does not include changes in areas other than valid pixels within the site. For sites other than Okhla, the difference between DCM and ASTER is generally small, though the percentage of valid pixels in the DCM data is one of the factors that causes differences between the DCM and ASTER data. Moreover, for Bishkek, it should be noted that the DCM_2_ data were recorded after the latest ASTER observation image.

Okhla is still in operation, but because the biomining project started in July 2019, it is possible that the amount of waste deposited has decreased and the change value per day is negative. In addition, the valid pixel ratio within the DCM site has reached 94.5%, and the plot in Figure 8 shows that the ASTER values are highly variable around the target period (6524−7481), so the reliability of the ASTER regression equation may be somewhat low.

**Figure 9:**
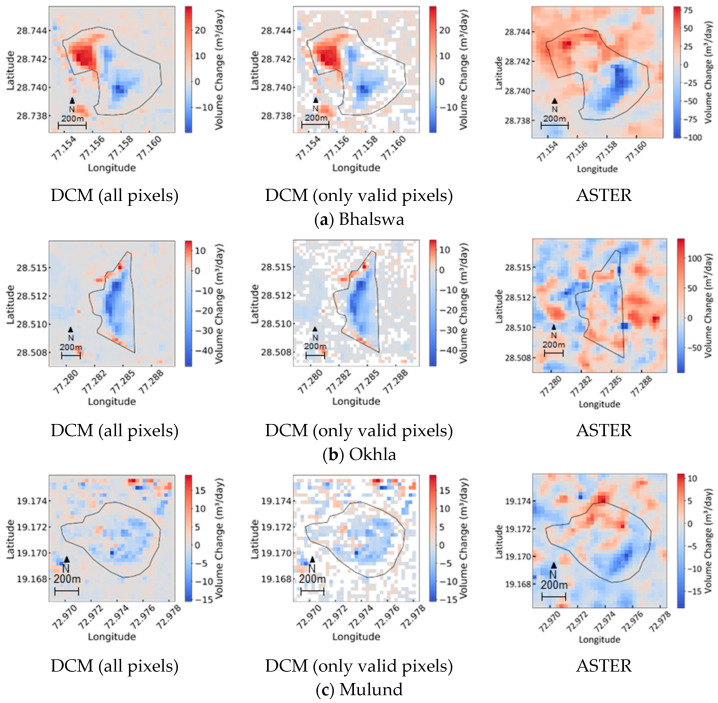
Daily volume change images for Bhalswa, Okhla, and Mulund using DCM data ((**left**): all pixels; **center**: only valid pixels) and those using ASTER images recorded near each DCM period (**right**).

Of the six sites, ASTER images were found for Bhalswa, Okhla, and Mulund that were observed near the observation times of DCM_1_ and DCM_2_. Figure 9 shows the daily volume change images for these sites using DCM and ASTER data. For the DCM, images with all pixels and images with only valid pixels (white indicates non-valid pixels) are shown. The ASTER images used were those observed on 15 May 2020 and 15 March 2021 for Bhalswa, on 5 November 2019 and 15 May 2020 for Okhla, and on 18 November 2018 and 12 November 2022 for Mulund. The consistency between DCM and ASTER data in the daily volume change images was the highest for Bhalswa and the lowest for Okhla, which is consistent with the results shown in Table 6.

## 4. Discussion

### 4.1. Reliability of Reported Values

In this study, we used values reported in various media, including academic papers and news reports, when converting the relative volume of waste measured by ASTER into absolute volume and when evaluating the absolute volume obtained in this way. There are various ways of obtaining such values. For example, the reported value for Mulund in 2015 in Table 4 is the result of a contour survey. The reported value for Dhapa (active site) in 2014 was calculated assuming that the landfill site was a truncated cone. The reported values for Dhapa (active site), Dhapa (closed site), and Dhapa (active + closed sites) on 1 October 2009 were calculated based on aerial photographs obtained from Google Earth and AutoCAD software. The reported values for Al Wafaa and Al Amal were calculated using a numerical integration approach based on direct current resistivity (DCR) sounding and borehole data. On the contrary, in many cases, the methods used to obtain these values and their accuracy are unclear, and some of them may include irregular information obtained by visual inspection. As mentioned above, many landfills in developing countries are poorly managed, and there are problems such as the lack of a weigh station to determine the weight of trucks at the landfill or the fact that the weigh station is not always in operation. For example, in a report from SCS ENGINEERS, it is reported that “*Actual disposal rates at the landfill are uncertain prior to activation of the truck scale in 1996, and significant amounts of wastes generated by the City reportedly were disposed at other sites prior to 1998. In addition, during the site visit the scale did not appear to be actively used to record incoming truck weights*” [83]. In addition, although the amount of waste brought in and the amount of waste accumulated at the site are reported in news articles and reports by the United Nations and other organizations, almost none of this information does specifies the exact observation period or observation method. In addition, there are many sites where such information does not exist. In some cases, there are large differences even between values reported for the same landfill site on close dates. Therefore, even when there are reported values, it should be noted that their reliability is uncertain. These problems with reported values of waste indicate the need for technology to monitor waste volumes in a unified and objective manner using the same method across sites and demonstrate the usefulness of the method using the DCM and the method used in this study.

### 4.2. Comparison with Method Using DCM

ASTER, which was used in this study, is an optical sensor, so it is affected by weather. This problem is particularly disadvantageous for monitoring in humid tropical regions. Contrarily, waste monitoring using the DCM has the advantage of not being affected by weather because it uses SAR, but at present, the problem is that the target period is short, from 2016 to 2022, and it is limited to the analysis of volume changes within this period. In addition, two different DCM products are required for the analysis of the amount of change, but only 6 of the 15 sites met this requirement. Furthermore, of the six sites that met the requirement, the average percentage of valid pixels that could be analyzed was 70%, and it was difficult to analyze the entire area at many sites. In addition, the fact that only the amount of change between two points in time can be analyzed, and that the estimation error of the amount of change becomes large when the time between two points is short, such as less than 50 days, are also problems. However, these problems result from the DCM product not yet being fully developed, so they will be resolved in the future as the DCM product is enhanced.

When comparing the results obtained using DCM and ASTER, they were relatively consistent. This is because both are satellite measurements, and both analyze the amount of waste based on the shape of the sediment.

### 4.3. Uncertainty of ASTER Estimates

Since the resolution of ASTER DSM images is 30 m, it is difficult to perform a detailed analysis based on the shape of the landfill. However, in this study, bias correction and statistical approaches are introduced to suppress the error of each estimated value, and since the smallest Dhapa (closed) site has an area of approximately 177 pixels, it seems that the use of the ASTER DEM product is appropriate for the purpose of analysis on the order of 10^5^ to 10^6^ m^3^ on a total volume basis.

The time-series plot of the ASTER-based relative volume of waste, shown in Figure 8, shows some variations at some sites. As shown in Table 3, the RMS of the regression residuals exceeded 1.5 (×10^6^ m^3^) at four sites (Deonar, Dhapa (active), Al Wafaa and Al Amal, and Jam Chakro), with the maximum value being 6.509 (×10^6^ m^3^) at the Al Wafaa and Al Amal site. The possible causes of these variations include the errors in the ASTER DEM product. The maximum elevation error in the product is considered less than 15 m. At first, we expected that the factors causing these errors would be the cloud cover, the pointing angle of the sensor, and the satellite’s orbit number, but in our preliminary survey, we were unable to clearly confirm the effects of these factors. In fact, since the ASTER DEM products are generated through stereo matching, their error characteristics may be affected by changes in surface patterns, atmospheric scattering conditions, etc., as well as by the matching parameters, and a detailed analysis focusing on the error characteristics of the ASTER DEM product is necessary. Therefore, in this study, we focused on how to mitigate errors through bias correction and statistical processing. This approach suppresses errors that are common to the entire site, but it does not suppress local variations within the site. The RMS of the regression residuals shown in Table 3 includes the effects of such local variations.

One of the factors that may cause such local variations is the state of waste accumulation at each site. Regarding sites with relatively high determination coefficients, the three sites in Delhi (Ghazipur, Bhalswa, and Okhla) had access roads for trucks bringing waste to the site, but as of 2017, the areas where the waste was accumulated formed large hills that were shaped like truncated cones or half-ellipsoids. At Mulund, the waste formed a circular pile, the access road extended from the entrance to the center of the site, and the truck routes formed a radial pattern. At Dhapa (active) and Dhapa (closed), the waste formed a semi-elliptical shape, and the access road was similar to that at Mulund. Oued Smar’s access road also had a similar shape to that of Mulund. However, although the determination coefficient for Pirana was over 0.9, the site had a shape with multiple scattered piles of waste. Kodungaiyur, with an R^2^ of 0.6752, was similar to Pirana. Jam Chakro, with an R^2^ of 0.6414, also had many access roads within the site, and waste was widely deposited across the entire site. Al Akaider, with an R^2^ of 0.6187, also had waste dumped evenly across the site, and no prominent hills were seen. Conversely, regarding sites where the determination coefficient was below 0.6, at Al Husaineyat (R^2^ = 0.2167), the garbage was dumped evenly in parts of the site, and no large hills were seen. At Al Wafaa and Al Amal (R^2^ = 0.4706), which were originally quarries, the garbage was dumped to fill in the preexisting holes. At this site, as of 2009, the access road extended from the north of the site to the center of the site, and from there, roads radiated outwards, with many hills formed within the site. At Bishkek, with an R^2^ of 0.1573, the waste was disposed of relatively gently, mainly on the western side of the site. From the above, it is thought that if the waste forms hills that are large enough compared to the resolution of ASTER, the volume of the waste can be accurately evaluated using the method in this study, but if small hills are scattered or the waste is deposited gently across the entire site, there is a possibility that this will cause large variations in some parts of the site. In addition, some sites experience fires caused by combustible methane gas and cigarette butts generated from landfill sites, and these fires are frequent in the summer when the temperature rises [116,117]. Smoke from these fires can be a factor in the error of stereo matching when generating DEM products.

Furthermore, for the purpose of understanding the increase or decrease in the amount of waste, it is thought that the use of time-series relative volume data obtained by applying bias correction to the ASTER time-series DSM images is sufficient, but when it is necessary to evaluate the total amount of waste or weight, some assumptions must be made, and errors associated with these assumptions will occur. For example, the method in Section 2.3.2 (1) assumes that the density does not change throughout the analysis period, but the density can vary depending on the composition of the waste, and the volume of waste deposited in the past can be reduced due to compaction, resulting in an increase in density. Although there are many cases where the amount of waste is reported in terms of weight, this uncertainty in waste density is a factor that causes errors when converting volume to weight.

In addition, at some landfill sites, the amount of accumulated waste may decrease due to biomining or fires, either intentionally or accidentally, and such decreases can be a source of error when converting relative volume to absolute volume using reported values of past waste amounts. In addition, there are cases where infrastructure facilities such as waste treatment plants, power generation facilities, and gas recovery facilities, as well as the homes of people who make a living by scavenging garbage (scavengers), are built within landfill areas. Although these structures are not waste, they can be a source of error when calculating waste volume using the method in this study. Furthermore, although the impact of short vegetation such as crops and grassland is small, there are areas in Oued Smar and Kodungaiyur where tall trees are thought to be growing, and at such sites, there is a possibility that errors will occur in the estimated relative and absolute volumes depending on the height and density of the trees and their changes. In addition, although the boundaries of each site were visually confirmed and set in this study, the waste disposal area may be expanded depending on the landfill site, so this should be taken into account when analyzing the data. Kruse et al. (2023) proposed a method for automatically extracting changes in waste disposal areas [118], and the automatic detection of areas using such a method is also promising.

In the calculation of absolute volume based on the initial topography of the site, as described in Section 2.3.2 (2), the validity of the initial topography calculated from the outer edge is an important factor that determines the accuracy. In developing countries, landfill sites and illegal dump yards are often located in slightly sunken areas, such as former water areas or former mining sites. There are some studies that have determined the initial topography through direct investigation using boring, electromagnetic surveys, and reference to past topographical maps, but the establishment of a method for accurately estimating the initial topography from satellite images and geographical data remains an issue to be resolved in the future.

## 5. Conclusions

Monitoring the amount of waste in open landfill sites, which are common in developing countries, is important from the perspective of building a sustainable society and protecting the environment. For some of these landfill sites, information such as the amount of waste deposited and the amount of waste brought in is made public in reports and news articles, but in many cases, the survey methods, timing, and accuracy are uncertain. In addition, there are many landfill sites for which such information is not available. In this context, monitoring waste volumes using satellite data is extremely useful from the perspectives of uniformity, objectivity, low cost, safety, wide area coverage, and simultaneity.

In this study, we conducted a time-series analysis of waste volumes at a total of 15 landfill sites using time-series DSM data from the satellite optical sensor ASTER, which has accumulated more than 20 years of observational data. Unnecessary variations seen between time-series images were reduced by bias correction using a reference area around the site. In addition, since all of the sites had been in operation before the first ASTER observation image, the analysis was based on the relative volume using the first observation image as a reference, but by utilizing various reported values, it was possible to convert the relative volume of the accumulation into the absolute volume and to convert volume into weight, enabling a direct comparison with the reported values. In this case, due to the lack of reliability of each reported value, it was not possible to indicate the accuracy of the ASTER-based estimates assuming that the reported values are true, so we adopted an approach of confirming their validity through comparison of the values. In addition, we also compared and evaluated the method of Louw et al. [44], which uses the DCM product developed by the satellite SAR TanDEM-X.

As a result, the method used in this study was found to be effective in capturing changes such as increases and stagnation in the amount of waste deposited at many sites, although there were some problems pertaining to a high degree of variability and reduced reliability at some sites due to the lack of waste deposition conditions and observation images. When compared with various reported values, some differences were seen, and while some of these were thought to be due to errors originating from ASTER, there were also cases where the errors were thought to be originating from the reported values. When compared with the analysis using DCM products, there was a relatively high degree of consistency, but as the DCM archive is not yet extensive, there were applicability issues, and as the average effective pixel ratio within the site was around 70%, there were issues pertaining to evaluating the entire site. However, the use of SAR, which is not affected by weather, is extremely useful for monitoring, especially in humid tropical regions. Therefore, in the future, we hope to see an integrated analysis combining data from optical sensors such as ASTER with data from SAR satellites such as TanDEM-X for waste monitoring. In this context, it is imperative to deliberate on the integration and analysis of data with disparate temporal intervals and accuracies to ensure the reliability of monitoring outcomes. Furthermore, as the development of satellite constellations with high-resolution optical sensors and SARs progresses, it is thought that it will be possible to monitor the amount of waste in open-type landfills in developing countries with high frequency and high resolution. It is hoped that the results of this study will be utilized in the field of urban planning and environmental conservation within developing countries, thereby contributing to the establishment of a sustainable society in the future.

## Figures and Tables

**Table 2:** Observation dates and times of the oldest and newest images of each site.

Site Name	Observation Date and Time	
	Oldest Image	Newest Image
Ghazipur	4 February 2001 05:54:02	5 May 2023 05:13:05
Bhalswa	4 February 2001 05:54:02	29 May 2023 05:13:02
Okhla	4 February 2001 05:54:02	5 May 2023 05:13:05
Deonar	27 March 2002 05:46:04	13 February 2023 05:34:04
Mulund	27 February 2001 06:02:04	25 January 2024 05:18:02
Dhapa (active site)	29 March 2000 05:10:02	5 April 2024 04:09:00
Dhapa (closed site)	29 March 2000 05:10:02	5 April 2024 04:09:00
Kodungaiyur	10 February 2004 05:16:03	26 May 2023 04:53:00
Pirana	6 March 2001 06:07:03	24 November 2022 05:39:04
Al Akaider (Al-Ekaider)	11 July 2001 08:36:02	18 August 2023 07:59:04
Al Husaineyat (Mafraq FDS)	1 May 2001 08:32:01	10 March 2024 07:50:04
Oued Smar	27 July 2000 10:51:04	19 August 2023 10:18:02
Al Wafaa and Al Amal (El-wafaa and El-amal)	10 August 2001 08:49:01	12 June 2023 08:13:03
Bishkek (BSWL)	26 May 2002 06:04:05	3 August 2018 05:58:04
Jam Chakro	28 October 2001 06:25:02	18 December 2023 05:45:01

**Table 3:** RMS of the regression residuals, maximum relative change during the ASTER observation period, and ratio of the former to the latter for each site.

Site Name	RMS of Residuals (×10^6^ m^3^)	Maximum Relative Change (×10^6^ m^3^)	Ratio
Ghazipur	0.861	5.715	0.15
Bhalswa	0.468	6.754	0.07
Okhla	1.009	5.107	0.20
Deonar	1.877	7.686	0.24
Mulund	0.764	5.486	0.14
Dhapa (active site)	3.890	8.380	0.46
Dhapa (closed site)	0.190	1.995	0.10
Kodungaiyur	1.129	4.409	0.26
Pirana	0.772	9.252	0.08
Al Akaider	1.015	4.125	0.25
Al Husaineyat	0.507	0.701	0.72
Oued Smar	0.967	10.34	0.09
Al Wafaa and Al Amal	6.509	19.38	0.34
Bishkek	1.406	1.824	0.77
Jam Chakro	2.906	7.337	0.40

**Table 4:** Comparison of ASTER-based volumes calculated using the two methods in Section 2.3.2 (1) and Section 2.3.2 (2) with the reported volumes on the specified date.

Site Name	Specified Date	Reported Value (×10^6^ m^3^)	ASTER-Based Value (×10^6^ m^3^) and Discrepancy with the Reported Value (%)		References
			Section 2.3.2. (1)	Section 2.3.2. (2)	
Ghazipur	1 January 2008	5.00	5.75 (+15.0%)	4.30 (−14.1%)	[89,90]
Bhalswa	1 January 2019	8.80	9.22 (+4.8%)	6.54 (−25.7%)	[89,91]
Okhla	31 December 2003	2.36	2.38 (+0.8%)	1.67 (−29.4%)	[89,92]
	31 December 2008	5.12	3.76 (−26.4%)	3.05 (−40.3%)	[89,92,93]
Deonar	1 January 2014	18.9–11.9	7.06 (−62.6 to −40.6%)	6.98 (−63.1 to −41.4%)	[73,94]
	1 January 2020	13.3–12.0	6.54 (−51.0 to −45.5%)	9.19 (−31.1 to −23.5%)	[62,94]
Mulund	1 January 2015	5.35	-	4.79 (−10.5%)	[95]
	6 October 2018	7.00	-	5.31 (−24.1%)	[96]
Dhapa (active site)	1 October 2009	3.54	-	2.62 (−25.9%)	[97]
	1 January 2014	62.7	-	4.05 (−93.5%)	[98]
Dhapa (closed site)	1 January 2009	2.00	-	1.80 (−10.1%)	[66]
	1 October 2009	2.28	-	1.89 (−17.2%)	[97]
Dhapa (active + closed sites)	1 October 2009	5.79	5.51 (−4.8%)	4.90 (−15.3%)	[97]
Kodungaiyur	1 September 2021	6.40	-	6.31 (−1.4%)	[99]
	18 October 2021	6.40	-	6.36 (−0.7%)	[100]
	10 May 2022	6.40	-	6.56 (+2.5%)	[101]
Pirana	1 January 2017	5.83–7.78	9.11 (+17.1 to +56.1%)	6.00 (−22.9 to +2.9%)	[83,102]
Al Wafaa and Al Amal	1 January 2021	42.3	-	24.3 (−42.6%)	[15]
Bishkek	28 August 2009	26.0	-	1.40 (−94.6%)	[103]

**Table 5:** Comparison of ASTER-based volume changes per day on the reference day and the most recent observation day with the reported amount of waste disposed of per day for ten sites.

Site Name	Reported Value (×10^3^ m^3^/day)	ASTER-Based Value (×10^3^ m^3^/day)		References
		Reference Day	Most Recent Day	
Ghazipur	1.67–2.08	1.35	0.0616	[62,90,104]
Bhalswa	1.25–2.67	1.46	0.198	[53,104,105]
Okhla	1.00–1.67	0.884	0.373	[53,92]
Deonar	3.00–7.58	1.01	1.01	[62,94,106]
Mulund	0.385–1.76	1.38	−0.0751	[107,108,109]
Dhapa (active site)	2.33–7.50	0.610	1.30	[84,96,98,110,111]
Dhapa (closed site)	2.92–16.1	0.671	−0.319	[97,98,111]
Kodungaiyur	1.82–5.14	0.186	1.07	[67,100,112]
Pirana	2.50–5.22	0.444	1.89	[83,113,114]
Bishkek	3.01–5.17	0.560	0.462	[103,115]

**Table 6:** Volume changes per day for DCM and ASTER data from DCM_1_ to DCM_2_ at the six sites. The DCM valid pixel rate is also shown.

Site Name	DCM Dates			DCM Valid Pixel Rate (%)	Daily Volume Change (×10^3^ m^3^/day)		
	DCM_1_	DCM_2_	Period (Days)		DCM	ASTER	Difference
Bhalswa	10 April 2019	30 July 2021	842	76.9	0.570	0.367	0.203
Okhla	16 December 2018	30 July 2021	957	94.5	−3.13	0.444	−3.57
Deonar	4 April 2019	7 July 2021	825	51.1	0.418	1.01	−0.589
Mulund	4 April 2019	7 July 2021	825	68.0	−0.465	0.159	−0.624
Kodungaiyur	4 March 2019	28 June 2021	847	56.8	0.858	0.926	−0.0681
Bishkek	19 July 2018	4 November 2019	473	65.0	0.855	1.47	−0.616

## Data Availability

Data are contained within the article.
